# Evolution of an Extended Pathogenicity Motif in VP2 of Infectious Pancreatic Necrosis Virus Isolates from Farmed Rainbow Trout in Turkey

**DOI:** 10.3390/v16060994

**Published:** 2024-06-20

**Authors:** Cuneyt Tamer, Kristina Ulrich, Nicholas Di Paola, Hanne Nur Kurucay, Harun Albayrak, Manfred Weidmann

**Affiliations:** 1 Department of Virology, Faculty of Veterinary Medicine, Ondokuz Mayıs University, 55139 Samsun, Turkey; cuneyt.tamer@omu.edu.tr (C.T.); hannenur.kurucay@omu.edu.tr (H.N.K.); 2 Institute of Aquaculture, University of Stirling, Scotland FK9 4LA, UK; ulrich_kristina@yahoo.com; 3 Center for Genome Sciences, US Army Medical Research Institute of Infectious Diseases, Fort Detrick, Frederick, MD 21702-5011, USA; nicholas.dipaola.civ@health.mil; 4 Medizinische Hochschule Brandenburg Theodor Fontane, 01968 Senftenberg, Germany

**Keywords:** infectious pancreatic necrosis virus, IPNV, Sp serotype, VP2, extended motif

## Abstract

Infectious pancreatic necrosis virus (IPNV) causes economic losses with a highly variable mortality rate worldwide, especially in rainbow trout. The virus has a double-stranded bi-partite RNA genome designated segment A and B. New complete genome sequences of nine rainbow trout isolates from Turkey were determined and subjected to phylogenetic analysis, identifying all as genotype 5 (serotype Sp). A time-dependent change in the extended pathogenicity motif of VP2 from P217T221A247 (PTA) to PTE P217T221E247 over a period of 10 years was identified. A wider analysis of 99 IPNV sequences from Turkey and Iran revealed the emergence of the motif PTE from 2007 to 2017, inducing significant morbidity in fry by 2013. In fact, displacement of the PTA motif, by the PTE motif in IPNV isolates appeared to be connected to a production peak of rainbow trout in 2013. An additional CAI analysis provided more evidence, indicating that rainbow trout culture in Turkey has an influence on the evolution of IPNV.

## 1. Introduction

Infectious pancreatic necrosis is a contagious viral disease affecting farmed saltwater and freshwater salmonid fish through vertical and horizontal transmission. *Infectious pancreatic necrosis virus* (IPNV) belongs to the genus *Aquabirnavirus* in the family *Birnaviridae* and has a bi-segmented (segment A and segment B) double-stranded RNA genome with approximately 6000 nucleotides. Segment A has a large open reading frame that encodes a polyprotein. The polyprotein, which is post-translationally cleaved, consists of four viral proteins in the following order: 5′-VP5, VP2, VP4, VP3-3′ [1]. VP5 partially overlaps with the 5′-prime region of the VP2 ORF and is a non-essential non-structural protein of variant size and unclear function and is not found in all isolates [2].

VP2 is the major capsid protein and contains a central variable domain and antigenic site (residues 183–335), as well as two hypervariable regions (residues 239–257 and 271–284) [3]. The central variable region includes a pathogenicity locus at positions 217 and 221 (PT persistent, PA, low virulent, TA virulent). VP3 is involved in activating the RNA-dependent RNA polymerase (RdRp) by binding to both RdRp and the dsRNA genome segments, but it is also involved in interferon alpha induction in salmon cells [4,5]. A VP2-VP3 fusion protein has been shown to induce a humoral response in immunized trout [6]. VP4 is a viral protease which is responsible for the post-translational cleavage of the polyprotein. Segment B has one large open frame (VP1) consisting of 2535 bp encoding the RdRp [3,4].

IPNV has been divided into 11 serotypes (Wb, Sp, Ab, He, Te, C1, C2, C3, Ja, TV-1, and MaBV) and seven genotypes (1–7). TV-1 and MaBV (genotype 7) are classified in Serogroup B. The other serotypes are classified as Serogroup A [7,8]. IPNV is distributed worldwide; common serotypes are Sp (genotype 5) and Ab serotypes (genotype 2) in Europe. The He serotype (genotype 6) and Te serotype (genotype 3) have been described in Germany and the United Kingdom, respectively [9]. The Wb (genotype 1), C1 (genotype 3), C2 (genotype 4), C3 (genotype 4), and Ja serotypes (genotype 5) originated in the USA and Canada [8,9,10,11,12].

In vitro studies indicate that reassortment in Birnaviruses can modulate IPNV virulence [13].

IPNV was first reported in Turkey in 2002 [14], and although it mainly caused asymptomatic infections in medium- and large-sized trout (>10 cm), it can, however, cause fatal outbreaks in fingerlings. IPN was a notifiable disease until 2007, and, initially, the demand to test for IPNV in outbreaks from farms was high, but it has dropped since 2007. Meanwhile, IPNV is considered enzootic in Turkey, and there has been no routine screening since 2017. A licensed vaccine is not in use; however, IPNV continues to be detected in sporadic samples received by the Virology Laboratory of the Faculty of Veterinary Medicine at the Ondokuz Mayıs University.

In our previous study, we described partial VP2 sequences of 62 IPNV isolates of serotype Sp from Turkey [15], confirming the observations reported by others [16,17]. In this study, we determined the whole genome of nine Turkish IPNV isolates by NGS. The overall analysis, including all IPNV sequences published from Turkey, indicates the emergence of IPNV isolates signified by previously described extended pathogenicity locus since 2007.

## 2. Materials and Methods

### 2.1. Viruses

Six isolates (Burdur 18, Aydın 21, Denizli 22, Tokat 24, Trabzon 27, and Kayseri 28) which were isolated during routine field screening surveys between 2005 and 2012 were kindly provided by Bornova Veterinary Control Institute/Izmir, Turkey (Gülnur KALAYCI and Buket ÖZKAN). IPNV Sdf-4 was provided by Central Fisheries Research Institute, Trabzon, Turkey (Hakan IŞIDAN). IPNV 1054 and IPNV GRE were isolated from dead fish sent to Ondokuz Mayıs University, Faculty of Veterinary Medicine, Virology Laboratory for virological diagnosis in 2013 and 2016, respectively (Table 1) [15].

### 2.2. Cell Culture

For cell culture isolation, under 1 g of fish whole body, with the tail and head discarded, were cut with a scalpel and were mixed with 1.5 mL of cold Leibowitz’s (L-15) medium (Gibco, Grand Island, NY, USA, Cat No: 11415-064). The mixture was homogenized for 1 min using a TissueLyser LT (Qiagen, Hilden, Germany). The homogenate was centrifuged at 1500× *g* for 30 min, and the supernatant was sterile-filtered (0.22 µm) and stored at −20 °C. Epithelioma papulosum cyprini (EPC) cells were used for the virus isolation. Briefly, EPC cells were cultivated at 25 °C in L-15 supplemented with 10% fetal calf serum (FCS), 1% penicillin/streptomycin (Sigma-Aldrich, St. Louis, MO, USA, Cat No: P4333-100ML), and 10 mM HEPES (Sigma -Aldrich, St. Louis, MO, USA, Cat No: H0887-100ML). A 1/100 dilution of the homogenates were inoculated onto 1-day-old EPC cells. The cells were maintained at 15 °C in a cooling incubator and checked daily for cytopathic effects (CPEs).

To check for bacterial infectious agents, the homogenates were also inoculated onto Tryptic Soy Agar (TSA), McConkey Agar (MCA), Cytophaga Agar (CA), and Shotts–Waltman Agar (SWA) and incubated at normal atmospheric conditions at 22 °C for 1–2 days (24–48 h).

### 2.3. Sequencing

Viral RNA extraction and RT-PCR were performed as described earlier [15]. After that, single-strand cDNA synthesis was generated using SuperScript™ III Reverse Transcriptase (Invitrogen, Carlsbad, CA, USA Cat No: 18080044) and ds cDNA was synthesized using the NEBNext^®^ Ultra™ II Directional RNA Second Strand Synthesis Module (New England Biolabs, Ipswich, MA, USA, Cat No: E7550L UK) following the manufacturer’s instructions, and ds cDNAs were cleaned by using RNAClean XP (Beckman Coulter, Brea, CA, USA Cat No: A63987).

Libraries were generated by using the Illumina Nextera XT DNA library preparation kit, and all the reads were conducted in a MiSeq sequencer (Illumina, Cambridge, UK) following a previously described protocol [17].

### 2.4. Phylogenetic Analyses

Whole-genome alignment was performed using Clustal W [18], and the bootstrapped phylogenetic tree of the whole-genome sequences was inferred using RAxML [19], both included in the MEGALIGN module of the DNASTAR software package, version 11. The neighbor net and parsimony net analysis were performed in SPLITS TREE 5.0 using a nucleotide sequence block of either the whole-genome segment sequences and from partial nucleotides sequences of segment A [20].

### 2.5. Codon Adaptation Index Calculation

To investigate if IPNV isolates from Turkey show evidence of codon adaptation to salmonid genomes, we calculated the codon adaptation indices (CAIs) for VP2. To calculate normalized CAIs, we first used the CAIcal program to obtain a “raw” CAI (rCAI). Next, an “expected neutral CAI” (eCAI) value was calculated by generating 1000 random sequences using a similar length, codon composition, and GC content. Normalized CAI values were then compared among different time points and viral lineages using a non-parametric rank test because the central tendencies trend varied throughout time more than each time point variance. To obtain our normalized CAI threshold, rCAI/eCAI values were calculated. Values greater than 1 were taken as evidence for codon adaptation to the reference set of codon preferences [21]. Values lower than 1 were taken as evidence that mutational bias is driving codon selection. The codon usage tables for *Oncorhynchus mykiss* and *Salmon salar* were downloaded directly from the publicly available Codon Usage Database (www.kazusa.or.jp/codon, accessed 25 April 2024).

## 3. Results

**Figure 1:**
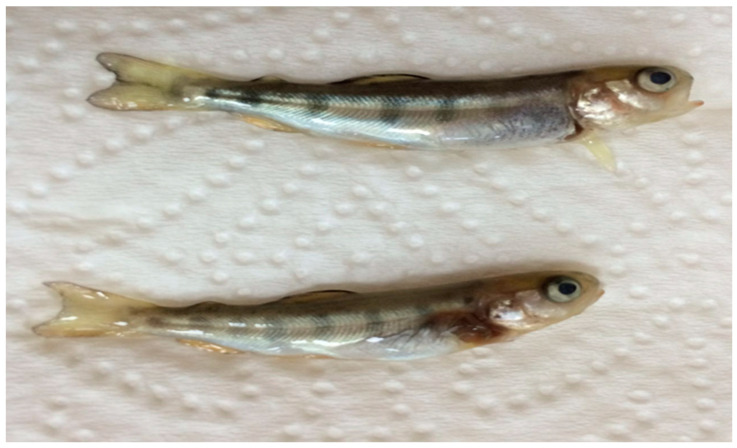
Gross pathology of rainbow trout fry with exophthalmia and spinal curvature from which IPNV 1054 was isolated. Size 4–4.5 cm; weight 0.7–1 g.

IPNV 1054 and IPNV Gre were isolated from rainbow trout fry of hatchery outbreaks from the Blacksea Region, Turkey, with fry showing abnormal swimming behavior close to the surface of the water. The gross pathology observed included exophthalmia, spinal curvature, skin darkening, and peritonitis with high mortality (Figure 1). All other isolates were isolated during routine screening from 1 g of asymptomatic rainbow trout.

### 3.1. Virus Isolation and Sequencing

**Figure 2:**
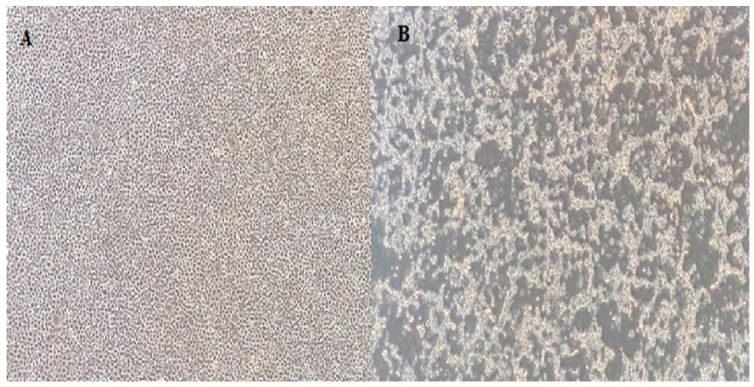
(**A**) EPC cell line control; (**B**) 7th day of inoculation of 1054 IPNV. Cells are visualized at the 40× magnification in an inverted microscope.

Nine isolates were successfully isolated from EPC cells, and all showed the typical IPNV CPE pattern (Figure 2). The RNA was extracted and subjected to sequencing. The main coverage of the genomes ranged from 20- to 40-fold. Assembled genome sequences were submitted to GenBank, and the accession numbers are listed in Table 1.

### 3.2. Phylogenetic Analyses

**Figure 3:**
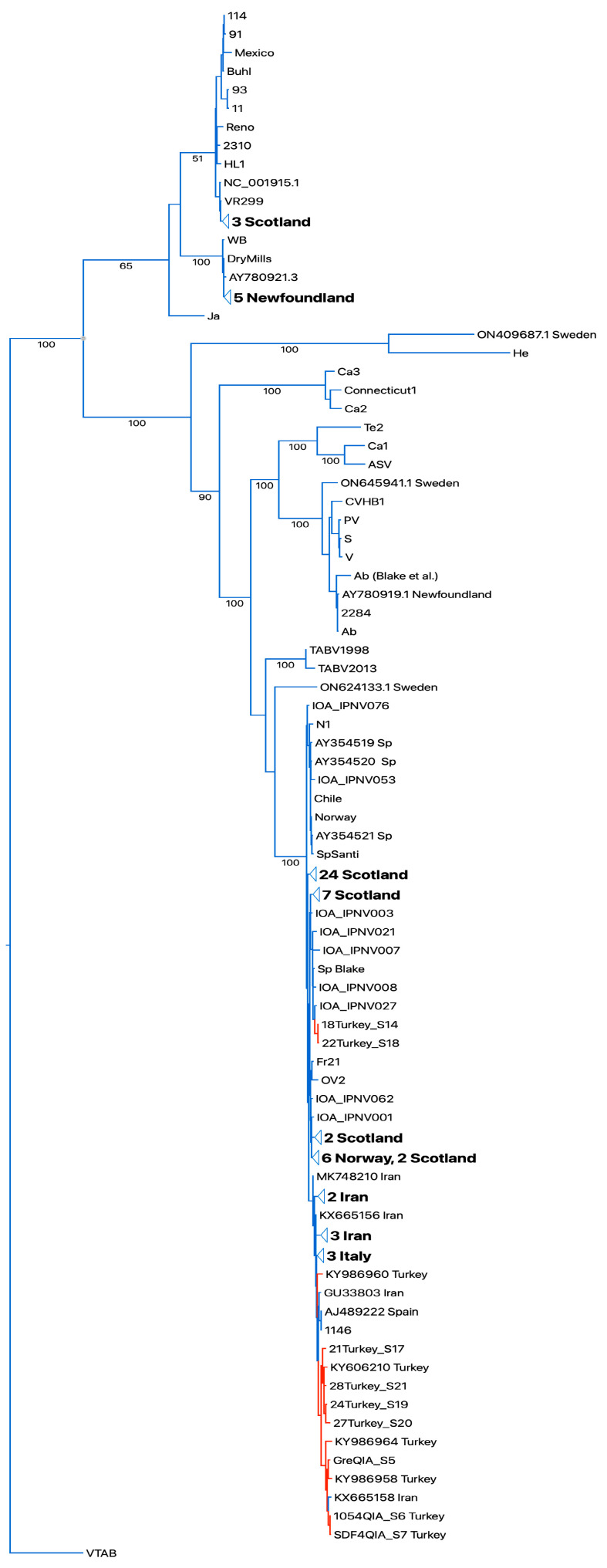
Bootstrapped RAXML tree of whole-segment A sequences. Sequences of Turkish isolates highlighted in red. Designations of IPNV isolates as in [22]. Bootstrap values on tree branches. Turkish IPNV isolates group in the Sp serogroup clade. TABV: Tasmanian aquabirnaviruses. Outgroup Victorian trout aquabirnavirus (NC_030242.1).

The segment A and segment B sequences were aligned with the respective available full segment IPNV sequences [16,22]. Both the Turkish IPNV isolate segment A and segment B sequences grouped in IPNV genogroup V (Sp serotype). For segment A, a set of two Turkish IPNV isolate sequences grouped with sequences of Scottish isolates in one subclade (segment A and B), and larger number grouped close to Spanish, Italian, and Iranian IPNV isolate sequences in a second independent subclade (segment A, Figure 3). Segment B, which codes for the RNA-dependent RNA polymerase, showed a similar grouping pattern (Figure S1).

Analysis of the extended pathogenicity locus of VP2 at amino acid positions 217, 221, and 247 [23] revealed that six sequences of isolates from 2005 to 2007 harbored the motif P217T221A247 (PTA), while three from 2010 to 2016 harbored the extended motif PTE P217T221E247 (PTE, Table 2).

**Figure 4:**
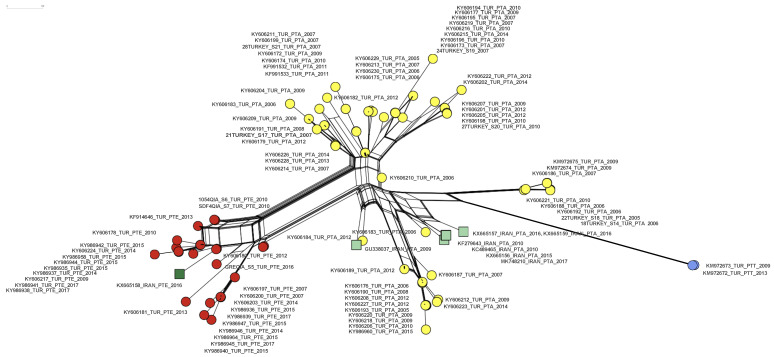
A neighbor net of 99 partial IPNV VP2 nucleotide set (405 characters from position 620 to 1025 (amino acids 170–303, respectively) of 91 isolates from Turkey ([15,16], this study), and of 7 isolates from Iran [24] (Table S1). Extended motif PTA: yellow circles (Turkey), light green squares (Iran). Extended motif PTE: red circles (Turkey), dark green square (Iran). Extended motif PTT: blue circles (Turkey).

A network analysis of a partial IPNV VP2 gene segment of IPNV trout isolates from Turkey and Iran published so far indicated two distinct emergence events of the PTE motif in Turkey from 2007 to 2017, representing roughly 30% (27/91) of the sequences described. One of the events is connected to the emergence of the PTE motif in Iran. Additionally, a PTT motif emerged in Turkey in 2009 in 2/91 sequences (Figure 4).

**Figure 5:**
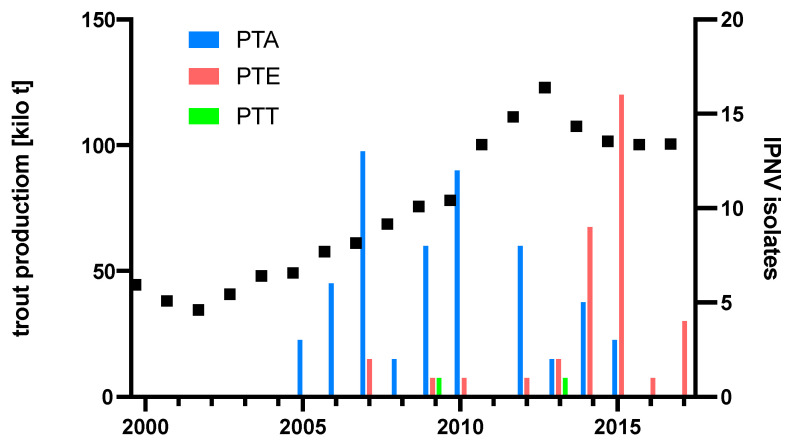
Production of rainbow trout in Turkey and pathogenicity type of diagnostic IPNV isolates. Production figures [25]. Total 101 isolates: 62 from [15], 30 from [16], and 9 from Table 1.

To understand if the emergence of the PTE over time might have been influenced by rainbow trout production levels, diagnostic IPNV isolates and their respective PTA and PTE motives were plotted over time, and rainbow trout production tonnage in Turkey was added. The resulting plot indicates the displacement of PTA isolates by PTE isolates from 2014, preceded by a production peak in 2013 (Figure 5).

**Figure 6:**
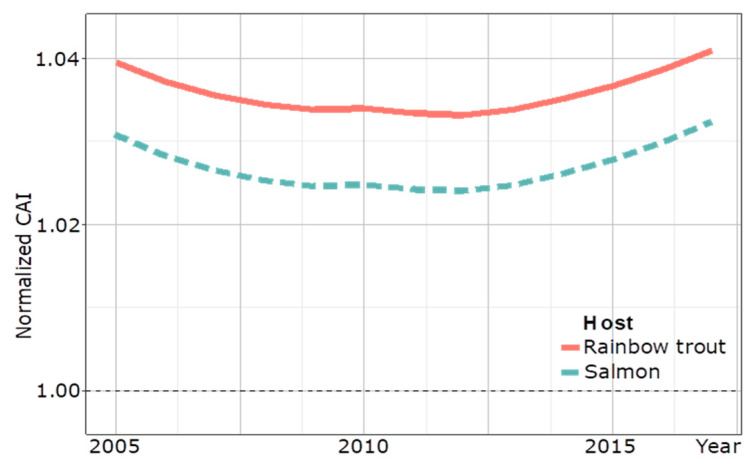
Temporal CAI analysis of 89 partial VP2 amino acidic sequences (169 amino acids) of IPNV isolates from Turkey to the host genome of rainbow trout from 2005 to 2017. The CAI curve for salmon is a control. A dotted line at 1.00 marks the delineation between evidence for adapted and un-adapted host-specific codon usage.

To assess the extent of the adaptation of IPNV to the host over time, a codon adaptation index (CAI) of a partial VP2 amino acid sequence of the Turkish IPNV trout isolates calculated a surge of the CAI towards the trout genome trending to >1.04 between 2013 and 2017, whereas a decreasing trend of adaptation was seen before 2013 (Figure 6).

## 4. Discussion

Surveillance evidence from farmed and wild fish and cell culture work described the impact of amino acid changes at an identified pathogenicity locus in VP2 within the hypervariable region [26]. By using reverse-engineered infectious IPNV clones in infection experiments, it was eventually shown that the IPNV clones switched from non-virulent T217 T221 to high-virulent T217 A221 when the hosts were subjected to stress [27].

The idea that the pathogenicity locus P217 and A221 linked to virulence might be extended by A247 (PTA) was reported in field studies from various salmonids in northern Europe, which linked PTA variously to subclinical infection, or to low and high virulence in salmon [9].

Turkey and Iran reported the PTA motif from IPNV isolates of farmed rainbow trout. Given the fact of the vertical transmission of IPNV via fertilized trout eggs [28], an inadvertent importation of IPNV carrying the PTA motif is obvious, and IPNV carrying this motif has spread widely in Turkey and Iran, as suggested by the interleaved clades of the phylogenetic trees of IPNV isolate sequences from Europe, Turkey, and Iran [15,16,24] (Figure 3 and Figure S1). IPNV isolates with the PTA motif from Turkey have been experimentally shown to induce moderate virulence in rainbow trout [29].

This study focused on nine additional IPNV isolates from Turkey and complemented the existing IPNV sequence dataset now allowing to describe the gradual emergence of the PTE motif in IPNV isolates from 2007 to 2017. This is evident from the network analysis which suggests two independent transmission chains inside Turkey after the PTE motif evolved in 2007 (Figure 4, red circles). It additionally corroborates that IPNV carrying the PTE motif was most likely introduced to Iran from Turkey (Figure 4), as suggested earlier [24].

A switch from PTA to PTE occurred just after a peak in trout production in 2013 as the PTE motif dominated the diagnostic IPNV isolates from 2014 onward (Figure 5). This is reminiscent of the switch of IPNV serotype Ab to Sp, which occurred during the massive expansion phase of the Scottish salmon industry in the 1990s, concomitant with a significant increase in IPNV VP2 CAI to >1.05 [20]. A CAI analysis of a partial amino acid VP2 sequence of IPNV isolates from Turkey indicated that VP2 codon adaptation to rainbow trout in Turkey may only have started to surge after this production peak in 2013 to a CAI >1.04 by 2017, and it is to be expected that the analysis of future isolates would confirm this trend for the full-length VP2 sequence. Although the nCAI of IPNV isolates from Turkey suggests host-specific codon adaption since 2005, the initial decrease in nCAI likely resulted from other evolutionary factors, such as modulating gene expression to evade immune surveillance [30]. Notably, it is of concern that only the most recent PTE-carrying IPNV isolates of this study induced gross pathology; however, experimental evidence for the pathogenicity of the PTE motif carrying IPNV isolates has not yet been reported. Further characterization of IPNV–host dynamics is needed to help elucidate relationships between genotypes and phenotypes.

A recent analysis of IPNV isolated from diseased, vaccinated, and resistant freshwater salmon in Chile described eight configurations at VP2 positions 217, 221, and 247, which, however, do not include the PTE motif. All of these isolates showed evidence of pathogenicity in gross pathology, histopathology, and cell culture [31]. Our analysis additionally identified two IPNV isolates from a turbot and a rainbow trout carrying yet another motif (PTT), which apparently is rare but points to the fact that IPNV infecting seawater and freshwater salmonids can adapt the hypervariable region in the VP2 subject to influences in various natural and farmed environments. Evidently, IPNV is continually evolving in the rainbow trout culture setting in Turkey.

Turkey’s fish product exports and imports are worth USD 1.3 billion and USD 200,000 annually. In 2022, for example, 144,347 tons of freshwater rainbow trout and 45,454 tons of seawater rainbow trout were produced in Turkey. Thanks to the increasing demand, rainbow trout are exported to Europe and Russia [32]. Since 2007, IPN has no longer been a notifiable disease in Turkey, and, unfortunately, the requests for IPNV isolation and analysis have subsequently dropped significantly, and rainbow trout breeders now only request tests for fish diseases listed by the WOAH for fish exportation (such as VHSV, IHNV, and ISAV).

In light of the evidence presented here, the expansion of the rainbow trout industry since the described time window (until 2017) may have continued to foster conditions for the evolution of the VP2 pathogenicity locus; however, the absence of adequate surveillance will most likely impact on safeguarding fish health.

## Figures and Tables

**Table 1:** IPNV isolates and sequences derived.

	İsolate Name	Province	Fish Species	İsolation Year	Segment A and B Sequence ID	Accession No Segments A and Segment B	
1	18	Burdur	*O. mykiss*	2006	18 Turkey S14	PP777437	PP777446
2	21	Aydın	*O. mykiss*	2007	21 Turkey S17	PP777439	PP777447
3	22	Denizli	*O. mykiss*	2005	22 Turkey S18	PP777438	PP777448
4	24	Tokat	*O. mykiss*	2007	24 turkey S19	PP777441	PP777449
5	27	Trabzon	*O. mykiss*	2010	27 Turkey S20	PP777442	PP777450
6	28	Kayseri	*O. mykiss*	2007	28 Turkey S21	PP777440	PP777451
7	Sdf-4	Trabzon	*O. mykiss*	2010	SDF4QIA_S7	PP777443	PP777452
8	1054	Tokat	*O. mykiss*	2013	1054QIA_S6	PP777444	PP777454
9	Gre	Giresun	*O. mykiss*	2016	GreQI_S5	PP777445	PP777453

**Table 2:** Extended pathogenicity locus configurations of sequenced IPNV isolates.

İsolates	Years	VP2 aa Positions			Provinces	İsolation Sources
		217	221	247		
22	2005	P	T	A	Denizli (SW)	routine screening
18	2006	P	T	A	Burdur (SW)	routine screening
21	2007	P	T	A	Aydın (SW)	routine screening
24	2007	P	T	A	Tokat (BS)	routine screening
28	2007	P	T	A	Kayseri (C)	routine screening
27	2010	P	T	A	Trabzon (BS)	routine screening
Sdf-4	2010	P	T	E	Trabzon (BS)	routine screening
1054	2013	P	T	E	Tokat (BS)	outbreak–disease
GRE	2016	P	T	E	Giresun (BS)	outbreak–disease

## Data Availability

The sequence data or data used to construct the maps are available from the corresponding authors upon reasonable request. Representative sequences have been submitted to the NCBI GenBank database and will be released on 14 November 2024.

## Supplementary Materials

The following supporting information can be downloaded at: https://www.mdpi.com/article/10.3390/v16060994/s1, Figure S1: Bootstrapped RAXML tree of whole-segment B IPNV sequences. Sequences of Turkish isolates highlighted in red. Designations of IPNV isolates as in [24]. TABV: Tasmanian aquabirnaviruses. Outgroup Victorian trout aquabirnavirus (NC_030244.1). Table S1: Sequences used for network analysis.
