# Supplementary material for: Evolution of an Extended Pathogenicity Motif in VP2 of Infectious Pancreatic Necrosis Virus Isolates from Farmed Rainbow Trout in Turkey
Source: Viruses. 2024 Jun 20;16(6):994. doi: 10.3390/v16060994 (PMC11209135; doi:10.3390/v16060994)
Supplement: Supplementary file 1 [file viruses-16-00994-s001.zip › viruses-3033036-supplementary.pdf]

viruses MDPI

Figure S1 Bootstraped RAXML tree of whole segment B IPNV sequences. Sequences of Turkish isolates highlighted in red. Designations of IPNV isolates as in [24]. TABV: Tasmanian aquabirnaviruses. Outgroup Victorian trout aquabirnavirus (NC_030244.1).

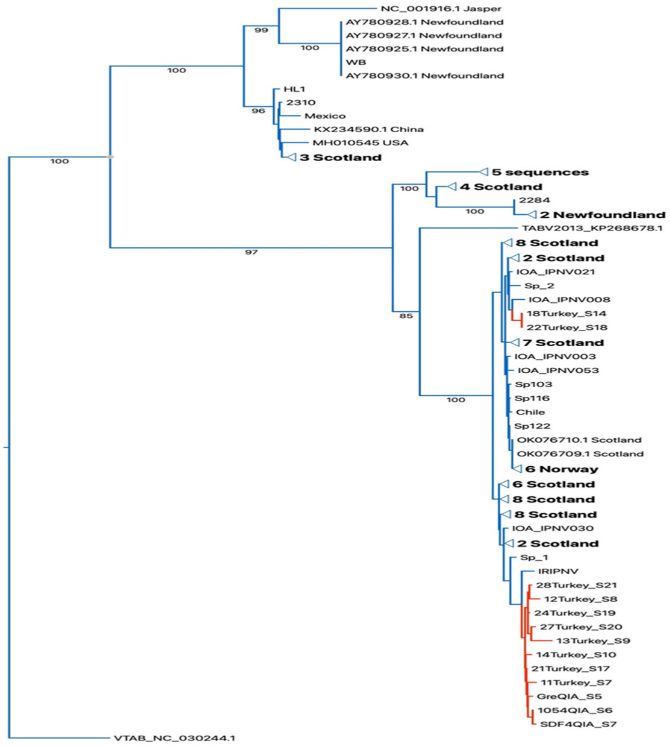

Table S1 Sequences used for network analysis

	Acession No / Seq-ID	Country	Pathogenic-ity motif	Year of Isolation
1.	22Turkey_S18	Turkey	PTA	2005
2.	KY606193	Turkey	PTA	2005
3.	KY606229	Turkey	PTA	2005
4.	18Turkey_S14	Turkey	PTA	2006
5.	KY606188	Turkey	PTA	2006
6.	KY606192	Turkey	PTA	2006
7.	KY606175	Turkey	PTA	2006
8.	KY606230	Turkey	PTA	2006
9.	KY606183	Turkey	PTA	2006
10.	KY606210	Turkey	PTA	2006
11.	KY606176	Turkey	PTA	2006
12.	KY606186	Turkey	PTA	2007
13.	21Turkey_S17	Turkey	PTA	2007
14.	KY606211	Turkey	PTA	2007

15.	28Turkey_S21	Turkey	PTA	2007
16.	KY606199	Turkey	PTA	2007
17.	KY606219	Turkey	PTA	2007
18.	KY606195	Turkey	PTA	2007
19.	KY606214	Turkey	PTA	2007
20.	KY606187	Turkey	PTA	2007
21.	KY606213	Turkey	PTA	2007
22.	KY606173	Turkey	PTA	2007
23.	KY606191	Turkey	PTA	2008
24.	KY606190	Turkey	PTA	2008
25.	GU338037	Iran	PTA	2009
26.	KM972675	Turkey	PTA	2009
27.	KM972674	Turkey	PTA	2009
28.	KY606204	Turkey	PTA	2009
29.	KY606209	Turkey	PTA	2009
30.	KY606207	Turkey	PTA	2009
31.	KY606220	Turkey	PTA	2009
32.	KY606218	Turkey	PTA	2009
33.	KY606172	Turkey	PTA	2009
34.	KY606212	Turkey	PTA	2009
35.	KY606177	Turkey	PTA	2009
36.	KC489465	Iran	PTA	2010
37.	KF279643	Iran	PTA	2010
38.	KY606221	Turkey	PTA	2010
39.	KY606198	Turkey	PTA	2010
40.	27Turkey_S20	Turkey	PTA	2010
41.	KY606194	Turkey	PTA	2010
42.	KY606206	Turkey	PTA	2010
43.	KY606196	Turkey	PTA	2010
44.	KY606174	Turkey	PTA	2010
45.	KY606216	Turkey	PTA	2010
46.	KF991533	Turkey	PTA	2011
47.	KF991532	Turkey	PTA	2011
48.	KY606182	Turkey	PTA	2012
49.	KY606184	Turkey	PTA	2012
50.	KY606227	Turkey	PTA	2012
51.	KY606208	Turkey	PTA	2012
52.	KY606205	Turkey	PTA	2012
53.	KY606201	Turkey	PTA	2012
54.	KY606189	Turkey	PTA	2012
55.	KY606222	Turkey	PTA	2012
56.	KY606179	Turkey	PTA	2012

57.	KY606228	Turkey	PTA	2013
58.	KY606215	Turkey	PTA	2014
59.	KY606226	Turkey	PTA	2014
60.	KY606223	Turkey	PTA	2014
61.	KY606202	Turkey	PTA	2014
62.	KX665156	Iran	PTA	2015
63.	KY986943	Turkey	PTA	2015
64.	KY986960	Turkey	PTA	2015
65.	KX665157	Iran	PTA	2016
66.	KX665159	Iran	PTA	2016
67.	MK748210	Iran	PTA	2017
68.	KY606197	Turkey	PTE	2007
69.	KY606200	Turkey	PTE	2007
70.	KY606217	Turkey	PTE	2009
71.	KY606178	Turkey	PTE	2010
72.	SDF4QIA_S7	Turkey	PTE	2010
73.	KY606180	Turkey	PTE	2012
74.	1054QIA_S6	Turkey	PTE	2013
75.	KY606181	Turkey	PTE	2013
76.	KF914646	Turkey	PTE	2013
77.	KY606203	Turkey	PTE	2014
78.	KY986946	Turkey	PTE	2014
79.	KY606225	Turkey	PTE	2014
80.	KY606224	Turkey	PTE	2014
81.	KY986937	Turkey	PTE	2014
82.	KY986940	Turkey	PTE	2015
83.	KY986964	Turkey	PTE	2015
84.	KY986947	Turkey	PTE	2015
85.	KY986936	Turkey	PTE	2015
86.	KY986935	Turkey	PTE	2015
87.	KY986942	Turkey	PTE	2015
88.	KY986958	Turkey	PTE	2015
89.	KY986944	Turkey	PTE	2015
90.	KX665158	Iran	PTE	2016
91.	GreQIA_S5	Turkey	PTE	2016
92.	KY986945	Turkey	PTE	2017
93.	KY986939	Turkey	PTE	2017
94.	KY986938	Turkey	PTE	2017
95.	KY986941	Turkey	PTE	2017
96.	KM972673	Turkey	PTT	2009
97.	KM972672	Turkey	PTT	2013
